# The Week's Work

**Published:** 1910-09-24

**Authors:** 


### The Week's Work.

### SETTLEMENT OF THE WEIR CHARITY.

The recent extraordinary general meeting of the Governors of the Bolingbroke Hospital, Wandsworth Common, has come to a decision which virtually brings to a conclusion the Weir charity dispute. The Governors, that is to say, acting on the advice of Sir Robert Finlay, have determined to repay the £5,000 which the trustees of the late Mr. Benjamin Weir in 1907 gave to the hospital, under the authority of the Charity Commissioners, towards the completion of the new wing. The dispute as to the employment of Mr. Weir's bequest has been discussed so often in our columns that there is no need to open the question now. It is sufficient to remind our readers that though the Bolingbroke Hospital actually lies in the borough of Battersea it receives more patients from Wandsworth, and this fact was brought forward when the Wandsworth Borough Council opposed the action of the Charity Commissioners in handing over to the Bolingbroke Hospital Mr. Weir's money. The return of that money puts an end to the dispute; but the important fact remains that this return cripples most seriously the funds of the Bolingbroke, which had been collected laboriously for its extension, the need for which no one denies. We hope that every step will be taken to make the public realise this, and that the action of the hospital in restoring a bequest to which other claimants asserted their right will bring a generous response from rich and poor, giving them indeed a further feeling of confidence in the Bolingbroke Hospital and its Governors.

### A MANCHESTER MAN'S CHARITIES.

Mr. A. Ahrens, of 100 Portland Street, and Demesne Road, Alexandra Park, one of Manchester's leading merchants, has celebrated his birthday by contributing large sums to local charities, and his example in this direction should prove inspiring to other rich men. As a thankoffering for having attained to three-score year and ten (having been born in Germany on September 16, 1840) Mr. Ahrens, who is a partner in the firm of P. Goldschmidt, and has been connected with the firm for forty-six years, has distributed £5,000 amongst ten institutions which are actively engaged in relieving the necessities of the sick and needy in Manchester. The charities which have benefited by Mr. Ahrens' munificence are: the Manchester Royal Infirmary (for the endowment of a bed to be called "The Adolph Ahrens Bed"), £1,000; the Manchester Victoria Memorial Jewish Hospital (for the endowment of a bed to be called "The Adolph Ahrens Bed"), £1,000; the Manchester Royal Eye Hospital, £500; the Ancoats Hospital and Ardwick and Ancoats Dispensary, £500; the Christie Hospital (Cancer Pavilion and Home), £500; the St. Mary's Hospitals, £500; the Manchester Hospitals for Consumption and Diseases of the Chest, £500; the Manchester and Salford Hospital for Skin Diseases, £200; the Home for Aged and Needy Jews and Jewish Shelter, £200; the Dental Hospital of Manchester, £100. The hospitals and many other charities in Manchester, as elsewhere, are badly in need of additional financial aid, and Mr. Ahrens' method of celebrating his birthday is to be heartily commended, and it is to be hoped that his generous example may be followed by other wealthy citizens. The steadily increasing work and need of our hospitals are constantly under the notice of the public, and there are many who prophesy that it will not be many years before it will be necessary to resort to rate-aid for their maintenance; but spontaneous generosity such as Mr. Ahrens has exhibited will encourage the committees responsible for their management to continue their efforts to maintain the voluntary system which has produced the perfectly equipped and up-to-date institutions we now have in our midst.

### THE SCHOOL CLINIC.

During the past eighteen months much discussion has been aroused on the subject of the medical inspection of school children and the problems involved in such inspection. One of the most important of these is the question of medical treatment. It is well known that the London County Council has lately increased its staff of medical school inspectors, and we may look forward to a considerable amount of excellent statistical material emanating from Dr. Kerr's department during the ensuing twelve months. But the Council has not further attacked the problem of treatment. The result of systematic inspection of the schools must be the elimination of a large number of children with certain lesions which imperatively demand treatment. Under the present system all that the school doctor can do is to point out to the parents the desirability of taking the child to a practitioner or to a hospital for treatment, and to give to such parent one of the usual cards stating that the child is suffering from an affection of some part of the body "which is likely to affect not only progress in school, but future prospects in life." This card, as is expressly stated, does not entitle the patient to treatment, but under the arrangements made with certain hospitals, a number of children are treated on certain fixed days. Anyone who has closely followed the working of this arrangement since the time of its inception must have been struck with the disadvantages it entails and the comparative ill-success that has attended it so far. In the circumstances it is easy to see that the arrangement is not popular, either with parents or with hospital staffs. The former have to wait sometimes for a very long time before their children can be attended to, and as a result they are disinclined to avail themselves of the system. The latter have an extra amount of work thrown upon their hands in an already overcrowded out-patient department, necessitating the appointment of extra clinical assistants and a rearrangement of out-patient hours. It is true the Council has voted special grants which allows of a slight remuneration to hospital officials who are specially told off to treat these children; but it is questionable whether it has acted in the best manner so far as both parties are concerned.

### THE CASE FOR SPECIAL TREATMENT.

It is every day becoming more apparent that the only way out of the difficulty is by the establishment of special school clinics, which would be worked in co-operation with the medical inspectors of schools. The exceptional success that has attended the Cambridge Dental Clinic is a proof of the practicability of such centres for treatment, and if one wanted further proof there are the Bradford, Strasburg, and York clinics, all of which are doing excellent work at a cost per patient considerably below that which is possible in a hospital out-patient department. From an economic point of view we are convinced that the establishment of such centres will be a benefit to the ratepayers, while the benefit to the children will be immense. Their establishment will, of course, mean an initial expense of several thousands of pounds, granting that the equipping and successful carrying on of an average clinic can be managed for a sum not exceeding £1,600, which seems to us a fair average. The whole case for the school clinic is admirably argued by Dr. Lewis Williams, Medical Superintendent of the Bradford Education Authority, in Dr. Kelynack's recently published book on "Medical Inspection of School Children," and those interested in the matter can hardly do better than read his illuminative chapter on the work that has already been done in his own district. From an institutional point of view such clinics are to be welcomed, since they will undoubtedly diminish the amount of routine work that now falls upon the out-patient staffs, more especially in certain departments, such as the eye, ear and nose, throat, and dental departments. Inevitably they will also tend to diminish the amount of adult out-patient work, and therefore in a measure help towards eliminating the abuse that now exists in this department of institutional work. The objections raised by general practitioners to the establishment of such clinics can be overcome by a little tact and commonsense, in neither of which the profession is deficient. As it is, the whole question is one which may well merit the attention of the British Hospitals Association. An expression of opinon on this matter emanating from so representative and influential a body of institutional experts, is likely to do more good towards stirring up local authorities to face the facts of the situation than any number of individual reports and recommendations from school doctors.

### THE IDEAL HOSPITAL.

The paper read by Dr. Goldwater on the "Problems of the Ideal Hospital" before the Medical Society of the county of New York during its latest discussion on the local hospital situation is an interesting one which will well repay study. After outlining Sir Thomas More's hospital scheme for the Utopian community—a scheme which is worth consideration to-day, especially so far as concerns hospital supplies and the need for strict regulation of foodstuffs offered to institutions by contract or otherwise—Dr. Goldwater paid a well-deserved tribute to Miss Nightingale's pioneering work with regard to sanitation, which has so immensely reduced the hospital mortality. He laid stress on the importance of looking upon the hospital primarily as a place for the care of the sick, not, as medical men are often in the habit of regarding it, as a medical school and research laboratory. Another interesting point brought forward for discussion was the question of economy. Dr. Goldwater holds, and in our opinion quite rightly, that it is a great mistake to compare hospitals on the basis of the cost of maintenance, and that the only true basis of comparison is the amount of work done. He referred to the high percentage of cost in private hospitals, where the quality of work was often far superior to that in public institutions. This comparison is not quite fair when applied to English and Continental hospitals, and it is an open secret that many private clinics and nursing homes are managed in a disgraceful way from many points of view Dr. Goldwater also attacked the huge problem of social work in connection with hospitals, which he held should be done in addition to, and not at the expense of, ordinary routine hospital work. He opposed the establishment of special hospitals, except in connection with general hospitals, a matter which is open to argument. With reference to the interesting question: "Should the medical staff be paid?" Dr. Goldwater agreed that it was necessary to give salaries to certain workers. The admitting physician—corresponding in some measure to our resident medical officer—the radiographer, and the pathologists should receive better pay than they do at present. "Whenever we ask a man to work for a hospital in a way that does not help him to build up a private practice he should be paid." That is Dr. Goldwater's decision, but it is open to very liberal interpretation, and does not help us much. What, after all, constitutes "help in building up a private practice"? And how long is the period of probation to be for the member of the staff to show that his appointment has failed to give him such assistance? There are many other interesting points in the paper, a summary of which appears in the current number of the *Medical Record.*

### THE BRITISH HOSPITALS ASSOCIATION CONFERENCE.

We would once more draw the attention of hospital workers to the forthcoming Conference of the British Hospitals Association, which takes place at Glasgow next week. The full programme and list of subjects for discussion were given in last week's issue of The Hospital. Those interested in the work of the Association and desirous of becoming members should lose no time in communicating with the Hon. Secretaries, Mr. West and Dr. Macintosh. The Conference, which is the first annual meeting of the Association, gives every promise of being a great success, and is sure to stimulate interest in the work of charitable institutions throughout the country. The more workers take part in it, and the discussion on the important subjects that figure on the agenda paper, the more representative will its meetings be and the more influential, therefore, any expression of opinion that comes from it. Every hospital worker who can possibly do so should make a point of attending the Conference.

### THIS WEEK'S DRUG MARKET.

THE improvement in business referred to in the report last week is maintained, and although the volume of business cannot be regarded as heavy, the prospects appear to be good. The expectation that refiners of glycerin would advance their prices has been fulfilled, and a further advance in price is by no means improbable. There is little change in the position of opium, and the higher price is well maintained; since the advance in the price of codeine, of 10d. per oz. there has been no further alteration in price, but if the value of opium continues to rise both morphine and codeine will most probably be dearer. The advancing tendency of the price of cod-liver oil continues. Cream of tartar and tartaric acid are again slightly dearer. Hydrastis is also dearer. The high price of jalap is maintained, and the drug continues to be in small supply. There is a fair demand for menthol, and higher prices are anticipated in some quarters. Potassium bromide and other bromides are unaltered in value, but the long expected advance is still looked for. Cubebs are slightly cheaper. Senega is also tending rather lower in value.

